# Anti-leishmanial effects of *Eryngium planum* and *Ecbilliun elaterum* methanolic extract against *Leishmania major*

**DOI:** 10.1186/s13568-023-01656-2

**Published:** 2024-01-03

**Authors:** Erfan Ghaderian, Bahman Rahimi Esboei, Parisa Mousavi, Maryam Pourhajibagher, Mohammad Mohsen Homayouni, Mohammad Zeinali

**Affiliations:** 1 Department of Parasitology and Mycology, School of Medicine, Aja University of Medical Sciences, Tehran, Iran; 2 Department of Parasitology, Toxoplasmosis Research Center, Mazandaran Registry Center for Hydatid Cyst, Mazandaran University of Medical Sciences, Sari, Iran; 3 Skin Diseases and Leishmaniasis Research Center, Isfahan University of Medical Sciences, Isfahan, Iran; 4 Dental Research Center, Dentistry Research Institute, Tehran University of Medical Sciences, Tehran, Iran; 5 Center for Communicable Diseases Management, Ministry of Health Treatment and Medical Education, Tehran, Iran; 6 Medical Parasitology, Department of Parasitology and Mycology, School of Medicine, Aja University of Medical Sciences, Tehran, Iran; 7 Department of Parasitology, School of Medicine, Mazandaran University of Medical Sciences, Sari, Iran

**Keywords:** Anti-leishmaniasis, *Eryngium planum*, *Ecbilliun elaterum*, *Leishmania major*, MTT assay

## Abstract

Leishmaniasis is a vector-borne disease, one of the most important neglected tropical diseases. Existing anti-leishmanial treatments are not effective for a long time and associated with toxic side effects so searching for a new, effective and safe alternative treatments against infectious diseases is greatly needed. This study is aimed to assess the leishmaniacidal effects of methanolic extracts of *Eryngium planum* (*E. planum*) and *Ecbilliun elaterum* (*E. elaterum*) on *Leishmania major* (*L. major*), In vitro. The selected plants were collected from northern areas of Iran. The methanolic extract from the aerial parts of plants were prepared using maceration methods. GC- Mass analysis was used to determine the compounds of the plants. Promastigotes of *L. major* was cultured in RPMI-1640 medium and the anti-leishmanial and cytotoxicity effects of extracts at concentrations of 100, 200, 400 and 800 µg/ml were assessed using MTT assay. The data obtained from gas chromatography revealed that α-Pinene, Caryophyllene oxide, β‐Caryophyllene, Bicyclogermacrene and α‐Bisabolol are the main compounds extracted from *E. planum* and α‐Pinene, Germacrene D, Caryophyllene oxide, γ‐Eudesmol and α‐Bisabolol are the main components of *E. elaterum*. The results of MTT Assay revealed that *E. planum* at concentrations of 800 µg/ml after 24 h at 400 µg/ml after 48 h and the *E. elaterium* at concentrations of 800 µg/ml after 48 h at 400 µg/ml after 72 h had similar anti-leishmanial effects to the positive control. These results indicated that *E. planum* and *E. elaterum* are the potential sources for the discovery of novel anti-leishmanial treatments.

**Supplementary Information:**

The online version contains supplementary material available at 10.1186/s13568-023-01656-2.

## Introduction

*Leishmaniasis* is a zoonotic parasitic disease caused by a parasite of the genus Leishmania in the family Trypanosomatidae, which is an obligate intracellular parasite (Yadav et al. 2023). Vertebrates, especially humans, dogs, hyrax, and rodents are the main hosts of this parasite. Leishmania parasites are transmitted to the host by the bite of sandflies, which are mostly of Phlebotomus and Lutzomia genera (Yurchenko et al. 2023). *Leishmaniasis* is a disease that is widespread throughout the world, particularly in tropical and subtropical regions. It is endemic in about 102 countries, with an estimated 12 to 15 million people affected globally. Around 1.5 to 2 million new cases and 70,000 dies were reported annually (Maia et al. 2023; Truong et al. 2023; Yurchenko et al. 2023).

This disease presents a wide range of clinical manifestations with potentially fatal outcomes and is classified into three types: Cutaneous *leishmaniasis* (CL), mucocutaneous *leishmaniasis*, and visceral *leishmaniasis* (VL) (Mann et al. 2021; Rodin and Smirnov 2020; Rojas-Jaimes et al. 2019). The CL has the highest incidence rate and more than 90% of these cases are reported in countries such as Afghanistan, Algeria, India, Iran, Syria, Saudi Arabia, Bolivia, Colombia, Nicaragua, and Brazil (Suprien et al. 2020; Volpedo et al. 2021). The CL manifestations is varied from asymptomatic and itchy papules at the bite site to malformed scars and serious physical damage to the person. Nodular, sporotrichoid, psoriasiform, verrucous, zosteriform, eczematous or erysipeloid lesions are the other atypical skin manifestations caused by CL. It has been proven that early diagnosis and treatment of the disease not only prevents the formation of malformed scars but also avoids the emergence of chronic forms of the disease (Garza-Tovar et al. 2020; Kumar et al. 2021).

Pentavalent compounds of antimony (Glucantime and Pentostam drugs) are the first and amphotericin B, pentamidine, and ketoconazole (for skin type) are the second line of drug choice for CL (Castro et al. 2023; de Vries and Schallig 2022) but, the use of these drugs has decreased due to their adverse side effects on the heart, liver, and kidney, as well as the painful injection and the drug resistance detected in recent years (Roatt et al. 2020). There are many drugs for the treatment of infectious diseases, one group of which is drugs extracted from plant compounds and their extracts, which are welcomed due to their low side effects; in this case, the effect of *Artemisia annua* on Malaria or the effect of Ivermectin on the *Strongyloides stercoralis* dangerous parasite (Ali 2023; Kshirsagar and Rao 2021).

*E. planum* is a native plant of Iran and is used in traditional societies of most countries as an edible vegetable, food flavoring, and medicine (Paun et al. 2019). The medicinal activity of *E. planum* is mainly due to the presence of triterpenoid saponin compounds, flavonoids (glycosyls of kaempferol and quercetin), and phenolic acids (rosmarinic, chlorogenic, and caffeic acids) (Shabani et al. 2022). Some of its medicinal activities include diuretic, anti-diabetic, expectorant, anti-inflammatory, analgesic, anti-fungal, and anti-amoebic (Arykbayeva et al. 2023). Also, it has been proven that the *E. planum* have satisfactory antioxidant properties due to the presence of high amounts of rosmarinic acid and chlorogenic acid (Kikowska et al. 2012). The *E. elaterium* is an herbaceous, perennial, and wild plant in West Asia and the Mediterranean area and is rich of Lipid, carbohydrates, various types of protein, triterpenoids (cucurbitacins) compounds, and cucurbitacins and their derivatives (including hexanorcocorbitation and glycosyl cucurbitation) (Razavi and Nejad-Ebrahimi 2010). In various research, anti-inflammatory effects (due to the production of IL-1, IL6, and TNFa factors) (Felhi et al. 2017a), cytotoxic (on gastric adenocarcinoma) (Bohlooli et al. 2012), antioxidant (Felhi et al. 2017b), antibacterial (on different strains of bacteria) (Felhi et al. 2017a, b; Hamidi et al. 2020), and antifungal (on *Candida albicans*) (Adwan et al. 2011) have been proven.

According to the excellent biological effects of *E. planum* and *E. elaterium*, as well as the importance of *leishmaniasis* disease and the problems in the use of current chemical drugs, this study aims to investigate the anti-*leishmanial* effects of these plants was designed and implemented on the *L. major* parasite.

## Methods

### Materials

MTT (3-(4,5-dimethylthiazol-2-yl)2,5- diphenyltetrazolium bromide) kit, Roswell Park Memorial Institute (RPMI)-1640 medium, Glacial acetic acid, Dimethyl sulfoxide (DMSO: 99%), inactivated fetal bovine serum (FBS), Novy-MacNeal-Nicolle (NNN) Medium and the penicillin– streptomycin solution (10,000 U/mL) were purchased from Sigma-Aldrich (USA). Novy- Fetal Calf Serum (FCS) purchased from Gibco. Methanol, Tween 80, Span 80, Span 60, stearic acid (SA), and solvents purchased from Merck Company. Distilled water was purified by a Milli-Q system (Millipore, Direct-Q).

### Plant collection and methanolic extraction

*E. planum* was collected from the plains of Babol city, Mazandaran province, and *E. elaterum* was collected from its three natural habitats in Ardabil province (Pars Abad, Angirlu, and Garmi). The genus and species of the plants and the species determination were identified by a pharmacognosy expert (Dr. Aroona Chabra), and herbarium samples were prepared, coded and stored in the pharmacognosy department of the Faculty of Pharmacy based on the APG system. After removing dust and waste materials, the aerial parts of the plant were separated and kept away from direct sunlight (due to the decomposition of anthraquinones) at room temperature. Then the plant was powdered and sieved to size no. 80, and 1.5 kg of plant powder was soaked in 3 L of methanol (Merck, Germany) for 48 h. After the incubation time, plant debris was removed by passing this mixture through Whatman filter paper (Kian Azma Teb, Iran) no. 1, and then the resulting solution was placed in a rotary evaporator (Pars Teb Novin, Iran) until all the solvent evaporated (Elmi et al. 2021).

### Gas chromatography and mass spectrometry

Agilent model 7120 gas chromatograph was used in this research. The capillary column of the device named 5MS-HP with a length of 30 m, a diameter of 0.25 mm and a film thickness of 0.93 μm was used. First, 0.9 µL of the sample was injected into the inlet of the device. At first, the inlet temperature reached 900 °C at a speed of 1 °C/min, then the device was placed at 30 °C for three minutes. After that, it was brought to 350 °C at a speed of 30 °C/min and kept for three minutes at this temperature. The detector of the gas chromatograph was 1-FID type, and helium gas was used as the carrier gas in this experiment at a rate of 9.9 ml/min. Agilent model 7120 device coupled with a 3273 C mass detector was used to identify essential oil components. The voltage of the device’s detector was 663.9 kV, and the device has the ability to register objects of 20 to 330 atomic mass units. The scanning speed of the device is 9.36 scans per second (Monadi et al. 2021). To identify the components of the essential oil, C8-C25 alkanes were first injected into the MS/GC device under the mentioned conditions, and then the inhibition time of each component was determined on the 5MS-HP column, and the Quats index of the compounds in the essential oil was calculated based on the relevant relationship and was compared with the values mentioned in reliable sources. In another method to prove the identifications, the main peaks of the mass spectrum of the unknown component sample of the essential oil were compared with the standard spectra provided by the device library and the name and structure of each component. It was determined using reliable sources. Also, additional investigations were done by matching the fragmentation patterns of mass spectra and Quats indices based on previous experiences from previous articles of our research group (Akbari et al. 2022; Al-Rubaye et al. 2017).

### Measurement of total phenol

The content of total phenol in the methanolic extract was measured according to Folin-Ciocalteu method. For this purpose, 0.5 ml of each extract with different concentrations was mixed with 2.5 ml Folin-Ciocalteu reagent diluted with distilled water at a ratio of 1:10. After 5 min, 2 ml of sodium carbonate (7.5% weight-volume) was added to the mixture, and after 15 min of storage in the dark, the absorbance of the samples was read using a spectrophotometer at a wavelength of 765 nm. The results were reported in terms of milligrams of Gallic acid per gram of dry weight (Akbari et al. 2022; Nikolova 2011).

### Preparation and culture of *Leishmania* parasite

Promastigotes of the standard strain of *L. major* parasite (MRHO/IR/75/ER) were purchased from the *Leishmaniasis* reference laboratory of the Faculty of Health, Tehran University of Medical Sciences and transferred to the NNN stock medium to grow sufficiently. In the next step, in order to multiply, the parasites are added to RPMI 1640 culture medium enriched with 15% heat-inactivated fetal calf serum (FCS), 1% streptomycin solution (50 µg/mL) and penicillin (50 U/mL), 5% CO_2_ at of 24$?$ (5). Promastigotes were used after three successive in vitro passages (de Sousa et al. 2023).

### Investigating the anti-*leishmanial* effect

In this study, the number of 1 × 10^6^ promastigotes of *L. major* in 100 µL of culture medium was added to each well of a 96-well plate. 100 µL of the concentrations of 100, 200, 400, and 800 µg/ml of the studied plants were added to the wells. Also, glucantime was used as a positive control and Phosphate-buffered saline (PBS) as a negative control. Then, the plate was placed in an incubator at 22 °C, and after 24, 48, and 72 h, using the MTT test and adding 100 ml of the MTT solution to each well, the survival rate of the parasites was read and evaluated by the amount of light absorption or OD of each well using the ELISA device, and the obtained information was recorded and checked (Mousavi et al. 2022).

### Toxicity study

J774 macrophage cells were obtained from Pasteur Institute, Karaj and cultured in RPMI-1640 culture medium enriched with 15% FCS and penicillin (100 µg/ml) and streptomycin (10 µg/ml) antibiotics. A number 1 × 10^6^ cells that were counted by a hemocytometer along with 100 µL of the culture medium was added to the wells of a 96-well plate in triplicate. Then 100 µL of each concentration (800, 400, 200, 100, and 50 µg/ml) of plant extracts was added to the wells and the plate was placed in the incubator at 22 °C. After 24 h, by adding 100 µl of MTT solution to these wells, the optical absorbance or OD of each well, which represents the survival rate of macrophages, was read with an ELISA reader and the rate of viability (%) was calculated by the following formula (Ghasemi et al. 2023):

$$Cell \; viability \; rate (\%)= \frac{OD \; of \; treated \; cells}{OD \; of \; control \; cells }\times 100$$

### Data analysis

As mentioned, all the tests were done in triplicate. The data obtained from this research were analyzed using SPSS version 22 and Graphpad Prism version 9.5.1 (733) statistical software, as well as ANOVA and T-test statistical tests.

## Results

### Gass chromatography and mass spectrophotometry

The data obtained from gas chromatography revealed that the Monoterpene hydrocarbons such as: α-Pinene (11.05%), β‐Pinene, Myrcene (2.65%), Limonene (3.14%), (Z)‐β‐Ocimene (0.81%) and Terpinolene (1.035%); Oxygenated monoterpenes such as: Camphor (5.05%), Terpinen‐4‐ol (0.92%), Myrtenol (1.34%) and α‐Terpenyl acetate (0.92%); Sesquiterpene hydrocarbons such as: β‐Bourbonene (0.023%), β‐Caryophyllene (2.31%), β ‐Copaene (12.54%), (Z)‐β‐Farnesene (0.4%), Germacrene D (0.4%) and Bicyclogermacrene (3.51%); Oxygenated sesquiterpenes such as: Caryophyllene oxide (8.45%), γ‐Eudesmol (14.35%) and α‐Bisabolol (4.5%), Phenolic compounds such as: Thymol (7.9%), Carvacrol (1.3%) and Eugenol (0.4%); Carbonylic compounds such as: 3‐Octanol acetate (0.3%) and Hydrocarbons (0.6%) such as: Nonacosane (0.2%) components are in methanolic extracts of *E. planum* and Monoterpene hydrocarbons such as: α‐Pinene (10.24%), β‐Pinene (1.05%), Myrcene (4.94%), (Z)‐β‐Ocimene (0.724%) and Terpinolene (0.586%); Oxygenated monoterpenes such as: Camphor (3.3%), Terpinen‐4‐ol (0.51%), Myrtenol (0.55%), Linalyl acetate (0.28%) and α‐Terpenyl acetate (0.54%); Sesquiterpene hydrocarbons such as: β‐Bourbonene (0.82%), β‐Caryophyllene (4.15%), β ‐Copaene (0.84%), Germacrene D (9.74%) and Bicyclogermacrene (3.61%); Oxygenated sesquiterpenes such as: Caryophyllene oxide (19.54%), γ‐Eudesmol (6.1%) and α‐Bisabolol (8.1%), Phenolic compounds such as: Thymol (0.9%), Carvacrol (0.6%) and Eugenol (0.3%); Carbonylic compounds such as: 3‐Octanol acetate (0.2%) and Hydrocarbons (0.21%) such as: Docosane (0.2%) and Nonacosane (0.2%) are present in the methanolic extracts of *E. elaterum* (Table 1).

**Table 1:** Composition (expressed in %) of the methanolic extracts of *E. planum* and *E. elaterum* using gass chromatography and mass spectrophotometry assay

Component	R	*E. planum*	*E. elaterum*	Identification	CAS No.
α-Pinene	945	11.05 ± 0.07	10.24 ± 0.021	RI, MS, Co-GC	1254-03-2
β-Pinene	958	2.65 ± 0.043	1.05 ± 0.02	RI, MS	1152-13-5
Myrcene	962	3.14 ± 0.07	4.94 ± 0.033	RI, MS	982-32-4
Limonene	974	0.81 ± 0.014	–	RI, MS	982-31-6
(Z)-β‐Ocimene	978	–	0.724 ± 0.019	RI, MS	982-30-2
Terpinolene	1021	1.035 ± 0.034	0.586 ± 0.025	RI, MS	215-14-2
Camphor	1125	5.05 ± 0.17	3.3 ± 0.047	RI, MS	215-32-1
Terpinen-4‐ol	1087	0.92 ± 0.024	0.51 ± 0.078	RI, MS	119-25-3
Myrtenol	1134	1.34 ± 0.064	0.55 ± 0.035	RI, MS	630-02-1
Linalyl acetate	1052	0.92 ± 0.032	0.28 ± 0.015	RI, MS	630-02-3
α-Terpenyl acetate	1134	0.023 ± 0.032	0.54 ± 0.022	RI, MS, Co-GC	630-02-8
β-Bourbonene	1066	2.31 ± 0.54	0.82 ± 0.034	RI, MS	6845-62-2
β-Caryophyllene	1087	12.54 ± 0.97	4.15 ± 0.67	RI, MS	11495-03-5
β -Copaene	1199	0.4 ± 0.044	0.84 ± 0.027	RI, MS	4856-05-1
(Z)-β‐Farnesene	1256	0.4 ± 0.004	–	RI, MS	13452-21-2
Germacrene D	1291	3.51 ± 0.054	9.74 ± 0.058	RI, MS	4125-05-4
Bicyclogermacrene	1341	8.45 ± 0.11	3.61 ± 0.087	RI, MS	3215-02-5
Caryophyllene oxide	1491	14.35 ± 0.94	19.54 ± 1.34	RI, MS	3215-41-3
γ-Eudesmol	1585	4.5 ± 0.054	6.1 ± 0.071	RI, MS, Co-GC	483-74-2
α-Bisabolol	1550	7.9 ± 0.92	8.1 ± 0.054	RI, MS, Co-GC	515-71-8
Thymol	1310	1.3 ± 0.021	0.9 ± 0.011	RI, MS, Co-GC	87-21-3
Carvacrol	1355	0.4 ± 0.011	0.6 ± 0.013	RI, MS	469-32-4
Eugenol	1425	0.3 ± 0.008	0.3 ± 0.011	RI, MS	458-21-8
3-Octanol acetate	1250	0.6 ± 0.014	0.2 ± 0.031	RI, MS	6750-52-5
Docosane	2200	–	0.21 ± 0.004	RI, MS	520-15-4
Nonacosane	2400	0.2 ± 0.01	0.2 ± 0.01	RI, MS	520-16-7

*Note*: *R* retention indices were defined relative to a series of n-alkanes (C8–C40) on capillary column VF5‐MS, *RI* identification made using the literature [Dario Kremer, 2021], *MS* identification made with help of database NIST02, Wiley 7 and homemade library, *Co–GC* identification using reference compounds, *CAS No.* CAS Registry Number [Dario Kremer, 2021]

### Total phenol

**Fig. 1:**
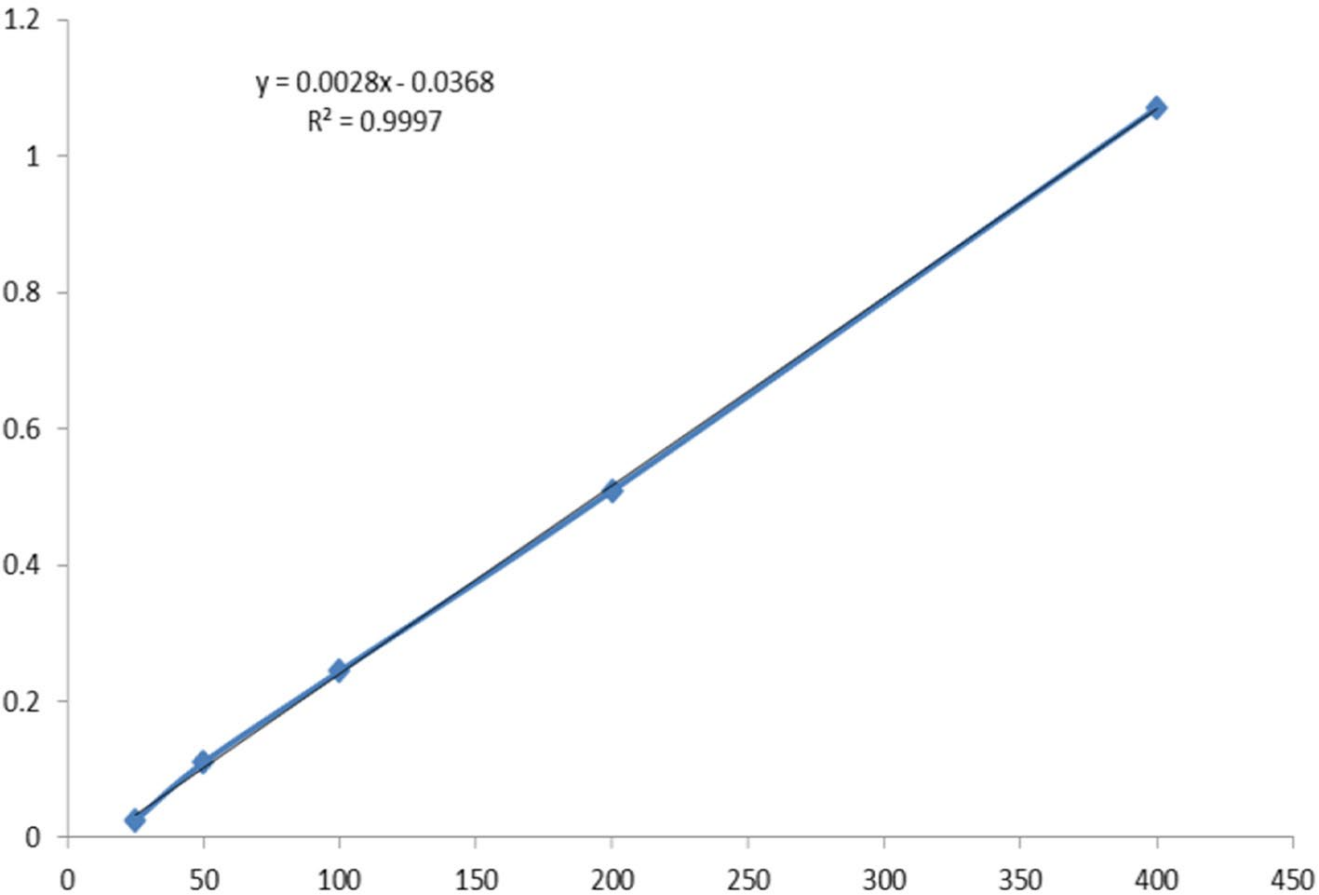
The standard curve of Gallic acid in different concentrations

The Folin-Ciocalteu assay method with the standard curve equation (y = 0.0061x + 0.0682, r2 = 0.9992) and expressed as Gallic acid equivalents was used to measure the total phenolic content of *E. planum* and *E. elaterium* methanolic extracts and a spectrophotometer instrument was used for reading the absorbance and the amount of total phenol were equivalent to 374.45 ± 5.235 and 297.88 ± 4.319 mg/g of dry extract, respectively (Fig. 1) (Supplementary data).

### Promastigotes cytotoxicity

The anti-leishmanial effectiveness of *E. planum* and *E. elaterium* at concentrations of 100, 200, 400, and 800 µg/ml was assessed in comparison to the negative and positive controls. The results exhibited that the anti-leishmanial effects of *E. planum* and *E. elaterium* were time and dose-dependent and significantly more effective than negative control (P < 0.05) (Supplementary data).

### The anti-leishmanial effectiveness of *E. Planun*

**Fig. 2:**
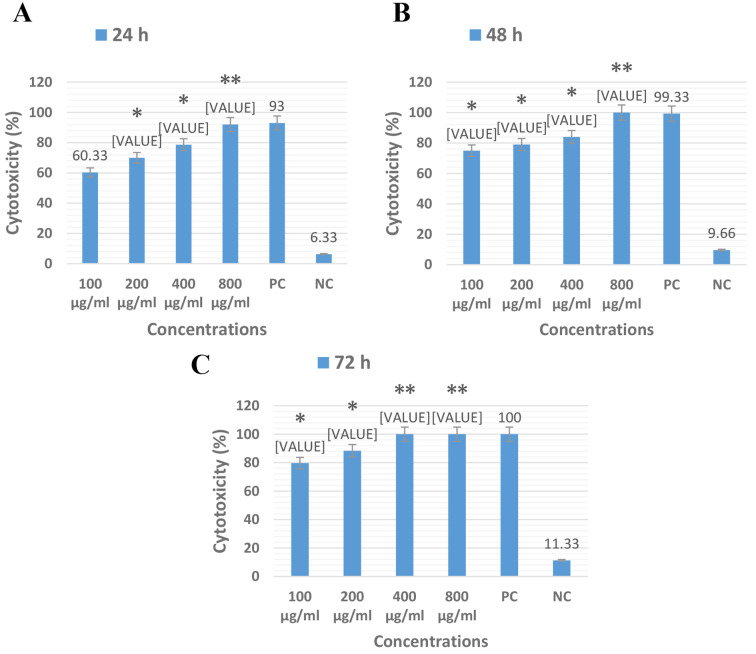
The cytotoxicity effects of the methanolic extracts of *E. planum* at concentrations of 100, 200, 400 and 800 µg/ml on promastigote stages of *L. major* after 24, 48 and 72 h in comparison of positive (PC) and negative (NC) controls

The results of the current study revealed that *E. planum* at all concentrations have significantly better effectiveness than negative control (PBS). The concentration of 800 µg/ml after 24 h and the concentrations of 400 µg/ml after 48 h had 100% anti-leishmanial efficacy on *L. major* promastigotes similarly to the glucantime after 72 h. The concentrations of 800 µg/ml showed better anti-leishmanial activity than positive control (glucantime) but this difference was not statistically significant (P < 0.05) (Fig. 2) Supplementary data.

### The anti-leishmanial effectiveness of *E. elaterum*

**Fig. 3:**
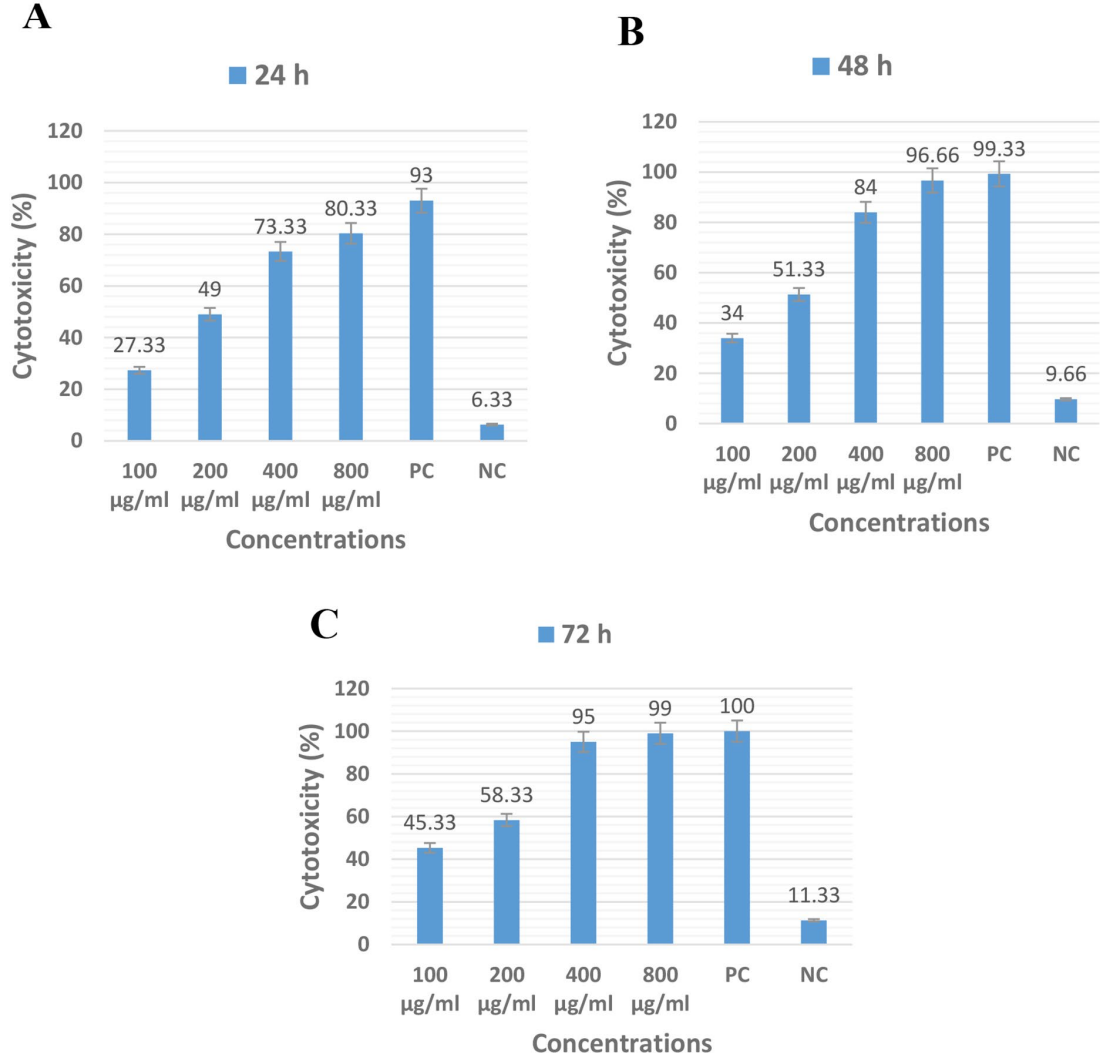
The cytotoxicity effects of the methanolic extracts of *E. elaterium* at concentrations of 100, 200, 400 and 800 µg/ml on promastigote stages of *L. major* after 24, 48 and 72 h in comparison of positive (PC) and negative (NC) controls

The anti-leishmanicidal efficacy of *E. elaterium* in all concentrations was less than the positive control but, the effectiveness of the concentrations of 800 µg/ml after 48 and 72 h and 400 µg/ml after 72 h was not significantly different from the glucantime as positive control (P < 0.05). The methanolic extracts of *E. elaterium* were more time-depended than *E. planum* (Fig. 3) (Supplementary data).

Our results indicated that the methanolic extracts of *E. elaterum* showed more anti-leishmanial effectiveness on *L. major* than E. *planun* but, this differences were not statically significant (P = 0.052).

### Cytotoxicity effectiveness on J774 cell lines using MTT assay

**Fig. 4:**
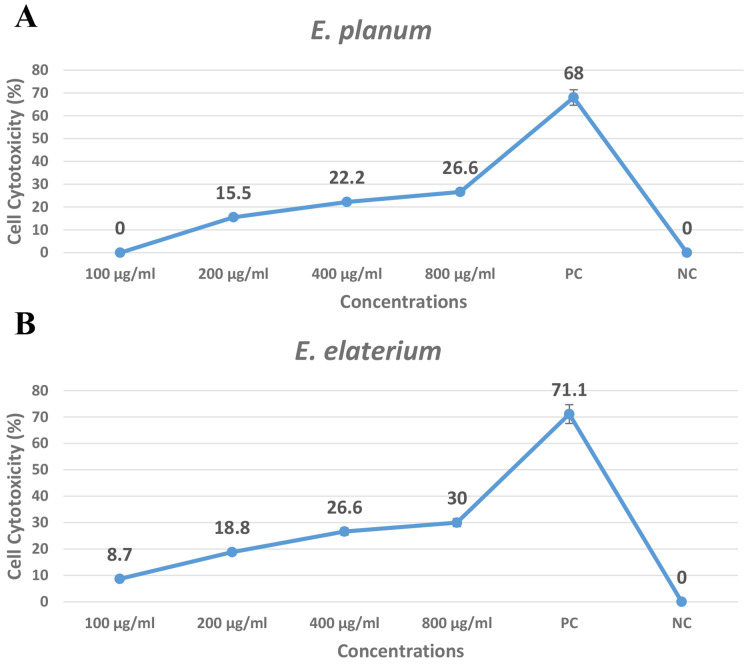
The cytotoxic effects of the *E. planum* (**A**) and *E. elaterium* (**B**) in concentrations of 100, 200, 400 and 800 µg/ml on J774 cell line in comparison to the Cis-platine (PC) and PBS (NC) as control groups

Beside the anti-parasitic effects, the cytotoxicity effects of *E. planum* and *E. elaterium* was assessed on J774 cell lines, and the results indicated that no toxicity effects was observed from both extracts. The extracts of *E. elaterium* and *E. planum* at highest concentration (800 µg/ml) showed significantly lower cytotoxicity effects than Cis-platine as positive control (P < 0.05) (Fig. 4) (Supplementary data).

## Discussion

In this study, the anti-*leishmanial* effect of *E. planum* and *E. elaterum* at concentrations of 100, 200, 400 and 800 µg/ml has been evaluated in vitro, and the results reported after 24, 48 and 72 h of exposure to *L. major* parasite. The results showed that the extracts of *E. planum* and *E. elaterum* had an acceptable anti-*leishmanial* effect, and the effectiveness of the plants increased with an increase in concentration and exposing time. Also, the extracts of *E. planum* and *E. elaterum* in all concentrations had a better effectiveness than the negative control group with a significant difference, and the statistical difference between the concentrations of 400 and 800 µg/ml with the positive control group was not statistically significant. Today, due to the side effects of chemical drugs and the high cost of preparing these drugs, the search for drugs based on natural compounds is a priority for the world’s health system practitioners (Dhama et al. 2023). Therefore, designing accessible, low cost, and safe therapies is essentially required. Natural products, due to the large structural diversity of secondary metabolites and novel chemical structures, conventionally play a crucial role in the exploration of new therapeutics and are valuable sources over centuries.

Also, some secondary metabolites produced in plants have the ability to protect plants against pathogens, insects, and other herbivores (Ozyigit et al. 2023). Therefore, plant extracts are investigated for their antibacterial, antifungal, antiviral, and anti-protozoal activities due to their active natural biological compounds(Dias et al. 2021). So far, extensive research has been done on the effect of different plant compounds on the *L. major* parasite in vitro and in vivo. Among the investigated drugs, *Artemisia aucheri* (Sharif et al. 2006) and *Camellia sinensis* (Rehman et al. 2022; Sharif et al. 2006) plants by Sharif et al., *Arnebia euchroma* and *Achillea plants* by Suzangar et al., zizyphusspina Christy by Albalawi et al. (Soosaraei et al. 2017), A. *Persica*, *A. fragrance* and *A. spicagara* by Najm et al. (2021) showed the best effectiveness.

Sharif et al. (2006) concluded that the concentration of 750 µg/ml of *Artemisia aucheri* plant extract causes the death of 25% of *L. major* promastigotes after 72 h (Sharif et al. 2006). Albalawi et al. (2021) showed that the IC50 of the methanolic extract of zizyphusspina Christy on *L. major* parasite after 24 and 48 h is 200 and 60 µg/ml, respectively (Albalawi 2021). Abed et al. (2009) investigated the effect of the aqueous extract of the *Fumaria officinalis* on the promastigote of *L. major* and showed that the highest inhibitory effect was related to the concentration of 4000 µg/ml (Abed 2009). Najm et al. (2021) proved that the IC_50_ of three different species of *Artemisia* sp. including *A. spicagara*, *A. Persica*, and *A. fragrance* are 51, 200, and 400 µg/ml, respectively, and the 50 cc of these extracts are 518, 560, and 700 µg/ml, respectively (Najm et al. 2021). By comparing the results of the above studies, it is clear that the plants used in this study have a better anti-parasitic effect on the *L. major* parasite in lower concentrations and times.

In recent years, plant compounds and active ingredient of plant are used to investigate the anti-parasitic and antimicrobial effects, which have been associated with better effects than existing chemical drugs. In a study conducted by Mousavi et al. (2022) on the anti-leishmanial effect of resveratrol and nanoemulsion of resveratrol on the promastigote of *L. major*, it has shown that the concentrations of 50 and 100 µg/ml of the mentioned compounds after 12 and 24 h, with a significant difference, have a more lethal effect than glucantim and amphotericin drugs as positive control groups(Mousavi et al. 2022).

In this article, we also investigated the anti-leishmanial effect of the methanolic extract of two plants, *E. planum* and *E. elaterium*, on the promastigotes of the *L. major* parasite in vitro. These two plants have been evaluated in the past for different medicinal activities due to their different chemical compounds. The plants used in this study, i.e., *E. planum* and *E. elaterium* have shown various medicinal effects due to their active medicinal compounds. The anti-*Acanthamoeba* effect of different compounds of the roots and leaves of the *E. planum* was investigated by Derda et al. (2013); the results showed that the flavonoid saponin compound isolated from the leaves of *E. planum* was the most effective at a concentration of 5 mg/ml and has inhibited 76% of trophozoites *of Acanthamoeba castellani* after 72 h (Derda et al. 2013). In the study by Al-Askar et al. (2023), the minimum fungicidal concentration (MFC) value of *the E. planum* on the *Fusarium solani* fungus in 48 h was 0.5 µg/ml while it was 1 µg/ml for Ketomonarol (Al-Askar et al. 2023).

In a study conducted by Dadar Talab et al. (2023) on the antibacterial properties of the methanolic extract of the *E. planum* on clinical isolates of *Escherichia coli* and *Staphylococcus aureus*, it was found that the effects of 1.2 mg/ml of the extract on human *Staphylococcus aureus* and 1.6 mg/ml on Staphylococcus aureus were significantly higher than gentamicin (Dadartalab and Nockghadam 2023). Paun et al. (2019) stated that *E. planum* is rich in polyphenolic compounds (flavonoids and phenolic acid), which have good antioxidant properties. They also stated that this plant has anti-inflammatory properties (inhibition of LOX and HYA enzymes) and improves type 2 diabetes due to the presence of polyphenol compounds and ursolic acid. As said, the *E. planum* has antimicrobial activity, which is due to the presence of phenolic compounds (Paun et al. 2019). The antimicrobial activities of polyphenols are due to effect on changes in the permeability of microbe membranes, changes in the stiffness of their membranes and cell walls, and changes in their internal activities due to the binding of phenols to their enzymes, so that rosmarinic acid and 3,4 dyhydroxyphenyllactic, which are considered phenolic compounds and are also present in *E. planum*, have biological properties, e.g., antioxidant, anti-fungal, anti-viral, anti-bacterial, anti-phlogistic, and anti-inflammatory activities. Also, chlorogenic acid, which is another phenolic compound found in this plant, has antioxidant, antiviral, antibacterial, anti-inflammatory and anti-allergic properties (Kikowska et al. 2012). Triterpenoid saponins is another important compound found in the root and leaves of the *E. planum* that has various biological effects. In a study conducted on animals, Francis et al. stated that these compounds have an antifungal effect (Francis et al. 2002). Some saponins and sapogenins have antiviral activity. Furthermore, saponins have a toxic effect on protozoa, which is widespread and non-specific, due to their detergent effect on the cell membrane of these organisms (Sharma et al. 2021).

According to past researches, *E. elaterium* has various phenolic compounds such as tannins, flavonoids, flavonols, and carotenoids. This plant has also lipid compounds and triterpenoids; each of these substances gives different medicinal properties to this plant. So far, various tests have been conducted on the medicinal effects of this plant; for example, Asgharian et al. (2021) stated that DCM and MeOH extracts of seeds and DCM, MeOH and N_hex extracts of *E. elaterium* root have significant antimalarial properties. Based on the previous experiments, the flavonoids and triterpenoids present in this plant create this property for this plant (Asgharian et al. 2021). Uslu et al. stated that the *E. elaterium* has anti-inflammatory properties due to inhibition of nitric oxide synthesizing enzyme (Uslu et al. 2006).

Adwan et al. (2011) investigated the antimicrobial effect of the ethanolic extract of the *E. elaterium* on Staphylococcus aureus and *Candida albicans* (*C. albicans*) strains and its effect along with penicillin G on *Staphylococcus aureus* strains; the results showed that, the minimum inhibitory concentration for the extract was 1.563 mg/ml, while this value was 1.758 mg/ml for penicillin G. Also, the minimum inhibitory concentration of this extract on *C. albicans* strains was 0.048 to 6.25 mg/ml, while this value for bifonazole was higher than 0.05 mg/ml, which indicates that this drug can also be useful in the treatment of this fungus (Adwan et al. 2011). One of the main problems that exist for many medicinal plants or traditional medicine is the lack of examination of the toxicity of these drugs. In this study, in addition to investigating the antiparasitic effect of methanolic extracts of *E. planum* and *E. elaterum*, the level of cytotoxicity of these compounds was also investigated. The results of cytotoxicity on the human macrophage cell line have shown that no toxic effects were observed, even at a concentration of 1600 µg/ml of the plants in question. Considering the very suitable anti-parasitic effects of *E. planum* and *E. elaterium*, as well as the absence of cytotoxicity in these plants, it is suggested that in future studies, the active ingredient of these plants such as saponins and flavonoids should be investigated separately, which can be used to determine the effects of these plants to predict the brilliance of these compounds. Due to the financial limitations in this study, the investigation of the anti-parasitic effect of these plants on the *leishmaniasis* wound caused by the *L. major* parasite in the in vivo phase has not been investigated. The results of the recent study have shown that *E. planum* and *E. elaterium* have acceptable anti-parasitic effects, and their effect increase with increasing time and drug concentration. Concentrations of 400 and 800 µg of the methanol extract of the studied plants were more effective than glucantime and amphotericin B. Also, in this study, it has been determined that *E. planum* and *E. elaterum* plants do not have any cytotoxicity even at a concentration of 1600 µg/ml.

### Electronic supplementary material

Below is the link to the electronic supplementary material.

**Supplementary Material 1:** The anti-leishmanial and cytotoxic effects of the E. planum and E. elaterium in concentrations of 100, 200, 400 and 800 µg/ml

## Data Availability

All data generated or analyzed during this study are included in this published article.
