# Supplementary material for: Anti-leishmanial effects of Eryngium planum and Ecbilliun elaterum methanolic extract against Leishmania major
Source: AMB Express. 2024 Jan 3;14:3. doi: 10.1186/s13568-023-01656-2 (PMC10764691; doi:10.1186/s13568-023-01656-2)
Supplement: Supplementary file 1 — Supplementary Material 1: The anti-leishmanial and cytotoxic effects of the E. planum and E. elaterium in concentrations of 100, 200, 400 and 800 µg/ml [file 13568_2023_1656_MOESM1_ESM.docx]

Table 2. The anti-leishmanial effects of *E. planum* at concentrations of 100, 200, 400 and 800 µg/ml after 24, 48 and 72 hours.

| Promastigotes Cytotoxicity | 24 h | 48 h | 72 h | *P- value^⃰^* | *P- value^⃰ ⃰^* |
| --- | --- | --- | --- | --- | --- |
| 100 µg/ml | 60.33±4.6 | 75.00±5.7 | 79.66±2.4 | .024 | .002 |
| 200 µg/ml | 70.00±1.4 | 79.00±5.0 | 88.33±1.6 | .004 | .000 |
| 400 µg/ml | 78.66±3.6 | 84.00±2.4 | 100.00±0.0 | .002 | .000 |
| 800 µg/ml | 92.00±2.1 | 100.00±0.0 | 100.00±0.0 | .225 | .000 |
| PC | 93.00±2.9 | 99.33±0.9 | 100.00±0.0 | - | .000 |
| NC | 6.33±0.4 | 9.6±0.9 | 11.33±2.0 | .000 | - |

PC: Positive Control (Glucantime), NC: Negative control (PBS). ^⃰^: P- value between treated groups and PC. ^⃰^ ^⃰^: P- value between treated groups and NC.

Table 3. The anti-leishmanial effects of *E. elaterium* at concentrations of 100, 200, 400 and 800 µg/ml after 24, 48 and 72 hours.

| Promastigotes  cytotoxicity | 24 h | 48 h | 72 h | *P- value^⃰^* | *P- value^⃰ ⃰^* |
| --- | --- | --- | --- | --- | --- |
| 100 µg/ml | 27.33±3.3 | 34±3.7 | 45.33±2 | .000 | .005 |
| 200 µg/ml | 49±0.8 | 51.33± | 58.33±2.4 | .001 | .001 |
| 400 µg/ml | 73.33±4.1 | 84±4 | 95±1.6 | .049 | .000 |
| 800 µg/ml | 80.33±3.8 | 96.66±2.8 | 99±1.4 | .422 | .000 |
| PC | 91.98±2.9 | 99.33±0.9 | 100±0.0 | - | .000 |
| NC | 6.33±0.4 | 9.66±0.9 | 11.33±2 | .000 | - |

PC: Positive Control (Glucantime), NC: Negative control (PBS). ^⃰^: P- value between treated groups and PC. ^⃰^ ^⃰^: P- value between treated groups and NC.

Table 4. The cytotoxic effects of the *E. planum* and *E. elaterium* in concentrations of 100, 200, 400 and 800 µg/ml in comparison to the Cis-platine and PBS as control groups.

| MTT assay-MQ Cytotoxicity | *E. planum* | *P- value^⃰^* | *P- value^⃰ ⃰^* | *E. elaterium* | *P- value^⃰^* | *P- value^⃰ ⃰^* |
| --- | --- | --- | --- | --- | --- | --- |
|  | Percent (%) |  |  | Percent (%) |  |  |
| 100 µg/ml | 0.0 | .000 | .000 | 8.7 | .000 | .000 |
| 200 µg/ml | 15.5 | .000 | .000 | 18.8 | .000 | .000 |
| 400 µg/ml | 22.2 | .000 | .000 | 26.6 | .000 | .000 |
| 800 µg/ml | 26.6 | .000 | .000 | 30 | .000 | .000 |
| PC | 68 | - | .000 | 71.1 | - | .000 |
| NC | 0.0 | .000 | - | 0.0 | .000 | - |

PC: Positive Control (Cis-platine), NC: Negative control (PBS)
